# Student acceptance of e-learning methods in the laboratory class in Optometry

**DOI:** 10.1371/journal.pone.0209004

**Published:** 2018-12-13

**Authors:** Monica L. Acosta, Aran Sisley, Jacqueline Ross, Ian Brailsford, Anuj Bhargava, Robert Jacobs, Nicola Anstice

**Affiliations:** 1 School of Optometry and Vision Science, University of Auckland, Auckland, New Zealand; 2 New Zealand National Eye Centre, University of Auckland, Auckland, New Zealand; 3 Faculty of Medical and Health Sciences, University of Auckland, Auckland, New Zealand; 4 Department of Anatomy and Medical Imaging, University of Auckland, Auckland, New Zealand; 5 Student Learning Services, University of Auckland, Auckland, New Zealand; 6 Department of Physiology, University of Auckland, Auckland, New Zealand; 7 University of Canberra, Bruce Campus, Australian Capital Territory, Australia; University of Botswana Faculty of Medicine, BOTSWANA

## Abstract

Today’s students have increased expectations for flexible learning options and evidence-based practice resources to be available to support curricular activities. We investigated: (i) the suitability of a static website for teaching ocular anatomy and physiology and an interactive version of the website with quiz and self-assessment activities and (ii) the usefulness of a blended online and in-lab environment to teach in Optometry. We administered a survey to compare responses of optometry students who had access to the interactive website, with those from students from a previous year who used the static version. We examined learning preferences of students in a focus group. Students were positive about the value of the website for their learning and the clarity of the website content. Nevertheless, objective comparison of pass rates for students using the static and interactive websites did not show significant changes. The majority of students commenting on the static website felt they did not get sufficient feedback via the website (67%) compared with only 22% from students who used self-assessments in the interactive website. Interestingly, users of the static website commented that it was perceived as just another resource while users of the interactive website commented on the usefulness of the material to review knowledge before laboratories. In the focus group, students reported they preferred a blended learning over the website alone even by students using the interactive website as they felt the need to revise content with the educator before the test. We conclude that there is acceptance of online learning methods due to the technologically ‘savvy’ environment of students in the first year of the Optometry programme but there is still dependence on the educator as the main administrator of their learning.

## Introduction

In today’s increasingly technologically-driven learning environment, it is important for an educator to consider how to reach students in the most effective way. Emerging technologies have enabled students to learn ‘on the go’, by retrieving resources which are accessible anytime and anywhere [1–3]. Online learning has become increasingly popular as it offers flexibility in terms of time, content control and the ability to tailor experiences to meet the learners’ personal objectives [4]. Learning at a tertiary level is traditionally the student’s own responsibility with the assistance of teacher-oriented facilitation during lectures, group activities, and group laboratory sessions. However, many students are used to an instructivist approach, where the educator acts as a ‘sage on the stage’, rather than a social constructivist approach where the educator acts as a ‘guide on the side’[5].

The University of Auckland Bachelor of Optometry (BOptom) programme is a five-year degree; students are selected either from a common Biomedical Sciences first year or via a graduate entry pathway for students who have completed an undergraduate degree in a related discipline. Students’ grades at the University of Auckland are expressed as a standard grading scale from 0 (fail) to 9 (A+) called the Grade Point Average (GPA) for each semester and a cumulative GPA for an entire programme. Grades achieved at external institutions are converted to Grade Point Equivalent (GPE) as there may be some adjustment made for inter-institutional grading differences. The entry requirement of the BOptom programme is a minimum GPA or GPE of 6.5 (equivalent to a B+). All students admitted into the BOptom degree have completed at least one year of university education and approximately 20% of students enter the BOptom programme via the graduate entry pathway. The Optometry programme is taught primarily through traditional face-to-face activities such as lectures, laboratories and tutorials with additional online study material available to students using the Learning Management System.

All Optometry degree programmes teach the fundamental optometric sciences including Ocular Anatomy and Physiology as a foundation for later learning of diseases of the eye and ocular therapeutics [6, 7]. There is a paucity of data on optimal ways for teaching this subject the content of which some students, especially those with little background in biological sciences, can struggle with. Only one previous study has specifically evaluated the role of e-learning in relation to ocular anatomy teaching [8]. In that study the authors developed both face-to-face interactive sessions complemented by five e-learning modules which significantly improved mean examination marks compared to previous student cohorts. However, in other areas of optometry teaching such as clinical skills teaching, e-learning in blended learning environments does not appear to improve students’ grades [9] and it is important to better understand in which situations within the optometry curriculum e-learning tools can best be utilised. More recently, e-learning tools used for general anatomy and physiology teaching have been shown to significantly improve student results [10], although the authors concluded that this was also associated with the development of students’ self-regulatory study skills which may have confounded results. As these factors can be difficult to tease apart, our study presented a unique opportunity to investigate the differences between an interactive website, which was specifically developed to promote deep learning through exercises and self-assessment tools, as compared with a static website which was primarily used for information exchange.

When designing e-learning tools for this higher education programme, not only the content but also the social, cognitive and teaching presence should be addressed [11]. Added equity considerations for University-entry have resulted in students entering health sciences programmes with diverse backgrounds and academic attainment levels [12]. We identified a need to enable successful outcomes of more able students while improving the quality and effectiveness of delivering basic anatomy and physiology concepts for all students. Regardless of the student’s background knowledge, the learning environment needed to be adequate for knowledge transfer of a specialist subject [13–15]. Practice-based approaches make a vital contribution to the dynamism and diversity of higher education [16, 17] and programmes that utilize multiple learning tools are more likely to have successful and positive learning outcomes [18]. Therefore, we saw an opportunity to integrate technology and to increase interest in the anatomy and physiology of the eye by simulating real evidence-based situations which require students to apply knowledge to understand the case.

In this study we investigated:

1. whether Optometry students with access to an interactive website on Ocular Anatomy and Physiology obtained better examination scores than students in the previous year who only had access to a static website;
2. students’ perceptions and impressions on the interactive versus static website approaches when combined with traditional face-to-face teaching approaches.

We conducted a survey to evaluate appreciation of the blended teaching approach by either users of the static or the interactive website and analyzed the answers.

## Methods

### Study period

At the time of studying Ocular Anatomy and Physiology, all students will have completed at least one year of University study. All students undertake the Ocular Anatomy and Physiology course in their first semester of study following admittance into Year 2 of the BOptom degree. The survey and focus group were conducted at the end of semester 2, 2011 and data analysis was conducted in 2012–2013.

### Study design

This research was a cross-sectional design using a mixed methods approach which allowed us to capture data from multiple sources (examination results, surveys and focus groups) in order to gain a more in-depth understanding of the impact of the interactive versus static websites. This blended learning environment consisted of online material and face-to-face teaching in a laboratory setting [19].

### Study process

Two versions of the online tool were investigated. The first was a static website as a student-focused tool to supplement the laboratory teaching environment. The blended learning environment allowed teaching through a portal that provided students with objectives, and resources for the delivery of basic concepts required for understanding the anatomy of the eye. The second version of the online tool was an interactive website with added areas for exercises, self-testing and assessments. This blended learning environment was also supplemented with face-to face laboratory teaching. We requested students’ perceptions and impressions about the websites and their learning experiences. The study was conducted with responses from 22 students that had access to the static website and 32 students that used the dynamic website.

### Design of the static and interactive websites

The static website contained descriptive information of the anatomy and physiology of the eye and was used and evaluated by students who had not experienced the interactive site and vice versa. The static website had referenced information about the anatomy and physiology of the eye and a link to a digital collection of histology images and a dissection video (the content of the site is explained in Table 1). The short dissection video-recording demonstrated key steps in a pig’s head dissection that students were required to undertake in a laboratory session. The same video was also available in the interactive website. In the static website it was the only movie activity while in the interactive version of the website there were several embedded movies showing videos for other laboratory topics such as anatomy of the skull, and cow eye dissection.

**Table 1:** Comparative description of the elements included in the websites.

	Static website	Interactive website
**Platform**	html	CourseBuilder
**Content**	Static pictures with legends	Interactive pictures with legends Interactive Hotspot pictures with legends Interactive diagrams
**Procedures**	Video for one laboratory activity	Videos for three laboratory activities
**Histology**	Digital collection *iViewer*	Digital collection *iViewer*
**Quiz**	none	Practice quiz at the end of each laboratory activity (20 in total) Clinical challenge scenario question
**Demo questions**	none	Laboratory test example questions
**Additional resources**	none	Students add online resources Students make their own assessment and answer questions using *Peerwise*
**View from students**	none	Comment box at the end of laboratory activities

Static website (first Year e-learning tools were introduced in the course Anatomy and Physiology of the eye in the Optometry programme) and interactive website (included improvements to the online tool that were accessed by a different group of students).

A collection of images of the Anatomy and Histology of the vertebrate eye was a resource that was available in both the static and interactive websites. This resource consisted of existing sections of ocular tissues mounted on glass slides that were imaged using a Leica DMR light microscope (Leica Microsystems, Germany) and converted to a web-suitable format. More than 2000 images of monkey and human eye structures were assembled using Adobe Photoshop software to re-construct the appearance of the whole eye. The interactive website contained the same information presented in the static site but had added wiki pages [20], multiple choice quizzes and links to online simulators (Table 1) that allowed students to appreciate levels of organization of tissues (cell to organ).

The interactive website was developed using "CourseBuilder", an e-learning tool developed by the University of Auckland that facilitated the addition of interactive resources [21] to the static website. A variety of interactive tools were used to stimulate more rapid learning and greater understanding by the students. These included animations of complex diagrams, short online quizzes at the end of each laboratory activity, additional embedded videos and animations relevant to other laboratory activities [22]. The interactive website included other additions generated and implemented by students. Students using the interactive version of the website were invited to create or identify new interactive learning and testing materials, and to embed external content that they deemed useful for their own learning. This meant that existing free-to use material was added as additional resources in the interactive website. The third addition to the interactive website also required student participation: once the website was completed, students were requested to use PeerWise [23], a resource developed at the University of Auckland to facilitate students’ participation in course development. Students were directed to develop their own questions and answers for the laboratory topics. The Optometry year-coordinator acted as the quality controller for the questions added through PeerWise, which then served as an additional self-assessment tool for the students (Table 1). The links that students added to the instructional material and offered through the interactive website were monitored by the course coordinator. Access to the interactive website was password protected and only available to the students that were currently enrolled in the Ocular Anatomy and Physiology course. Students that accessed the static website had completed the course at the time of the survey and there was no motivation for any student to re-access the website after the final assessment (final examination). The survey questions did not ask for a comparison between the static and interactive websites but rather used standard language to record students’ opinion about the e-learning programme based on their experience with it.

To comply with Institutional Review Board guidelines, the survey was completed anonymously but we did ask students to record whether they were in Year 2 (interactive website) or Year 3 (static website) of the BOptom degree. Those responses that did not identify the year of the course were not included in the analysis. At the time of the survey, none of the students had a need to access the material and we therefore think it is unlikely that any student accessed the website just to participate in this survey.

The effect that each of the static and interactive websites had on student learning was measured objectively by comparing laboratory test marks and final exam pass rates for the separate group of students using each site.

### Study population

One hundred and twelve students, from two consecutive years of the BOptom degree, were invited to participate in this study. While gender or age details were not recorded, the Optometry programme always includes a greater proportion of young (age 18–23 years-old) female (approximately 70%) students and we expect the same disproportion would apply to the survey respondents. A total of 54 students completed a survey on their impressions of an Ocular Anatomy and Physiology blended-learning website. Students in Year 3 experienced the static website version of the Anatomy and Physiology course the year before. Students in Year 2 had access to an interactive website the previous semester. The survey had the same questions and participants were directed to their experience with the e-tool during their time in the course. The invitation email was sent to all Year 2 and Year 3 students and resulted in 32 students from Year 2 and 22 students from Year 3 responding to the survey.

### Teaching modality in the laboratory class

As well as access to the website content, all students attended weekly face-to-face laboratory sessions for the 12-week duration of the Ocular Anatomy and Physiology course. The laboratory sessions were led by an academic member of staff with assistance from Teaching Assistants (one teaching assistant per 12 students). Each laboratory session was structured in a similar manner, with a 10-minute introduction followed by work in small groups and by laboratory questions and online exercises for students to complete individually during the class and at home. The academic staff member facilitated a short end-of-class question session to ensure students had covered all learning objectives prior to finishing the laboratory session.

### Delivery of the survey and focus groups

Ethical approval from the University of Auckland Human Participants Ethics Committee was obtained for the conduction of surveys and focus groups (2011/343). Questions from the standard University of Auckland Question Bank resource for assessment of the Optometry programme delivery were employed to construct the survey. Students responded on a five-point Likert-type or frequency scale strongly disagree (1), disagree (2) neutral (3), agree (4), strongly agree (5) and were instructed to select only one option for each question. Of the 55 students invited to comment on the interactive website 32 responded. Of the 57 students invited to comment on the static website 22 responded. The survey questions explored three specific activities: the suitability of the website for teaching ocular anatomy and physiology based on students’ impressions; the usefulness of the blended environment to teach in Optometry; and students’ expectations of a blended environment. Two methods were used to deliver the survey: an online form that was sent as an invitation to respond through students’ regular email and a paper-based form. To facilitate participation, the paper-based survey was delivered at the end of a routinely scheduled activity (scheduled lecture time) where snacks and drinks were available. Results were typed into an Excel table and the data was displayed in a graphical form.

Seven students accepted the invitation to participate in a focus group: four users of the static website and three users of the interactive website. The semi-structured focus groups were conducted by a qualified moderator who was familiar with the overall aim of the project but had no knowledge of the survey results and course content nor did he have access to the static or interactive websites. The moderator commenced the focus group sessions by asking broad general questions about the benefits and disadvantages of the website experiences after which the conversations were driven based on participants’ responses but the moderator ensured that the following topics were all addressed:

- what students liked or remembered about the version of the website the student had access to
- what scenarios in the laboratories stood out as being memorable
- what aspects of the scenarios were confusing or not particularly helpful
- what students remembered about the video laboratory
- the method of introduction and instruction of website use

### Data analysis

Student surveys were conducted anonymously which limited some of the demographic data we were able to collect, for example previous educational experiences, as this may have identified individual participants. This meant we were unable to analyse the association between previous educational experiences and the students’ perception of the website in this study and as such we are not able to comment on how demographic and educational diversity impacted on the students’ impressions of the website content.

Quantitative and qualitative methods were used to evaluate the effectiveness of the websites on student learning. The analysis included a comparison of the median and IQR for the Likert-scale responses. The average total mark and distribution of marks in the laboratory test was also considered. A plot of the distribution of marks for two key questions in the laboratory test (i) Students’ understanding of dissected eye structures; and ii) Students’ ability to identify a tissue shown in a digitalized histological section shown on both websites) was performed. A parametric test (Student’s t-test) was used in the comparison of average marks. Non-parametric tests (chi-square goodness of fit test) was applied in the comparison of the distribution of marks among users of the static website and interactive website. The hypotheses were:

1. Students who had access to the interactive website would have better final grades for the Ocular Anatomy and Physiology course than those students with accessed only to the static version;
2. The interactive version of the website would have a positive impact on student perception of online Ocular Anatomy and Physiology teaching.

The first hypothesis was tested by comparing the mean mark for the Year 2 cohort in Ocular Anatomy and Physiology versus the mean mark of the cohort before them.

Our second hypothesis sought to evaluate students’ perceptions of the interactive versus static versions of the website and therefore we employed independent thematic analysis of open ended commentary using a Grounded Theory approach to summarize key features of students’ impressions [24, 25]. Answers were categorized using a Likert scale, plotted as a diverging stacked bar chart and measured by comparing the percentage frequency distribution of agreement/disagreement. Discussions were recorded and transcribed verbatim and independently reviewed by three researchers. Each researcher was required to immerse themselves in the data through multiple readings of the material and initial note taking. Once this phase was complete, researchers undertook initial qualitative coding of the data to simply focus on specific characteristics of the data collected. This involved the researchers identifying important sections of the transcribed interviews and labelling them in relation to a theme or issue in the data. Once this initial coding had been completed, the three researchers met and common themes were identified through a modified Delphi approach. The validity of each theme was considered to determine whether it accurately reflected the data set. In some instances, there was not enough evidence to support individual themes, while in other cases large themes were further broken down into small sub-themes. Once each theme had been defined and named, the researchers conducted a detailed written analysis of that theme and considered how that theme fitted within the overall context of the study. Where possible, direct quotes from participants were used to articulate each of the themes identified by this process (Table 2).

**Table 2:** Qualitative analysis of open-ended comments from focus group participants evaluating static and interactive website versions.

Theme	Repeating ideas	Recommendations
What students remembered about the website	Online content, particularly videos, helped students prepare for laboratory activities and helped students when tutors were working with other learners	Monitor pre-class laboratory preparation e.g. student site access prior to face-to-face laboratory session
	Interactive diagrams in the website helped understand concepts	
What scenarios in the laboratories stood out as being memorable	Online quizzes helped students identify learning objectives and self-assess knowledge	Incorporation of further self-assessment quizzes into curriculum
What aspects of the scenarios were not helpful	Instructors still required to follow-up student questions	Add teacher-guided discussion at the end of the activity
What students remembered about video	Provided enough detail for students to undertake this task independently if tutor not immediately available	
Method of introduction and instruction of website use	Clear instructions still needed to understand program objectives	Teacher to confirm content before test

## Results

### Comparison of laboratory test answers and average total mark for the lab test

The first indicator of student progress considered was the average mark for two of the test questions directly related to the content delivered in the websites in consecutive years.

**Fig 1:**
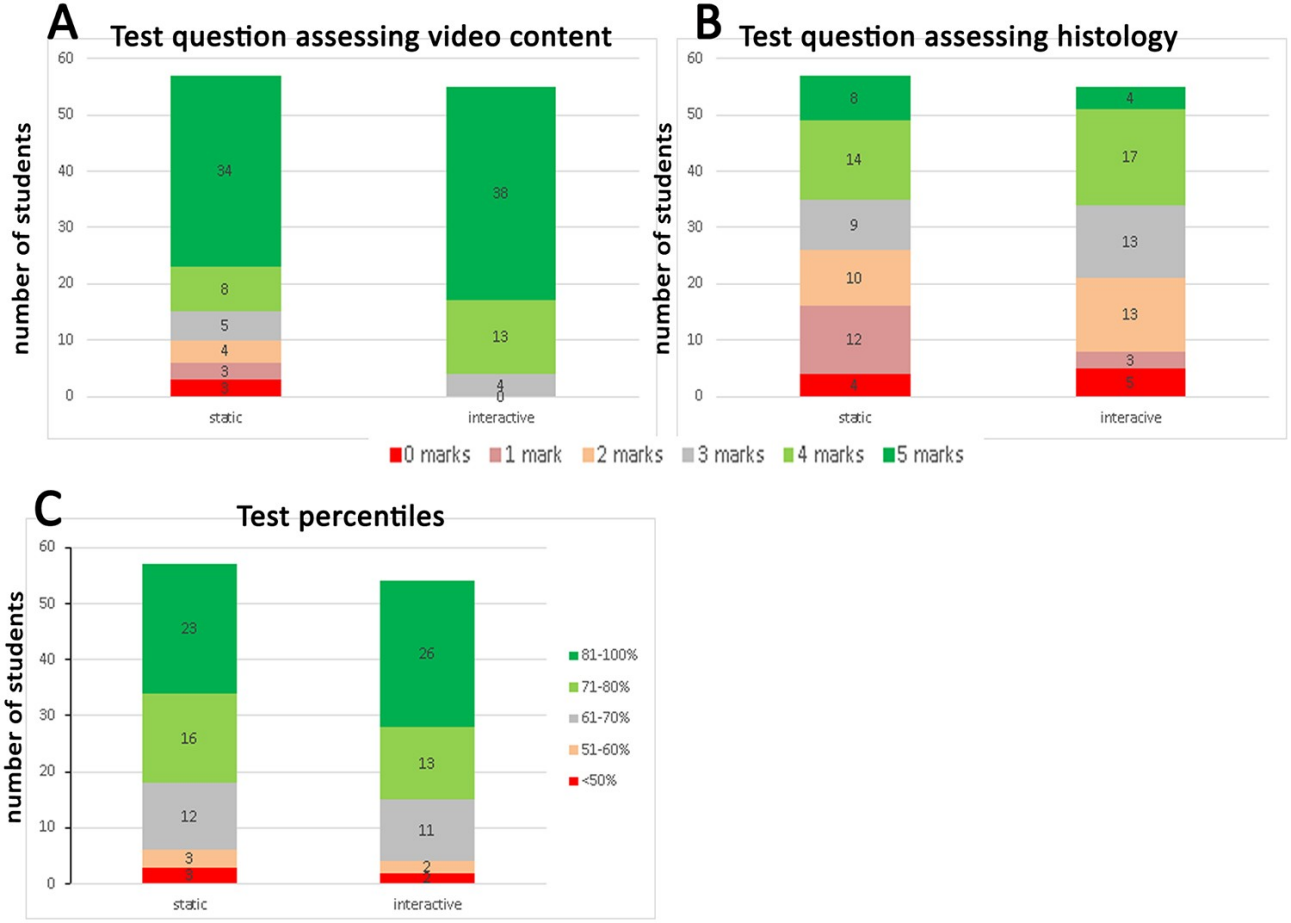
Distribution of test marks. The distribution of test marks for the examination question assessing content in the dissection video (A) and distribution of marks for the test question assessing histology of the eye (B). Overall marks obtained in the laboratory test grouped by percentile (C).

Users of the static website had a significantly lower mark (out of 5) than those with access to the interactive website (4.0 [± 1.5] vs. 4.6 [± 0.6], p<0.01). Conversely, the average mark (out of 5) for the question requiring students to identify histological sections was not significantly different between groups (2.8±1.4 versus 2.7±1.6 for users of the static site; p<0.05). Fig 1A shows that the distribution of marks for the question evaluating knowledge on dissected tissues was significantly different (p<0.05) but marks were more uniformly distributed and not significantly different for question 2 (Fig 1B). We found that the interactive laboratory website was also not a significant contributor to improvement in the total marks achieved in the laboratory test (Fig 1C). The average total mark for the class laboratory test (those that replied to the survey and those that did not) was 75.91 points ±15.9 (n = 57) while the average mark for the class with access to the interactive site was 79.0 ± 12.7 (n = 55). Non-parametric assessment of the distribution of marks for the laboratory test, did not show statistical significance (Fig 1C).

### Survey outcome

**Fig 2:**
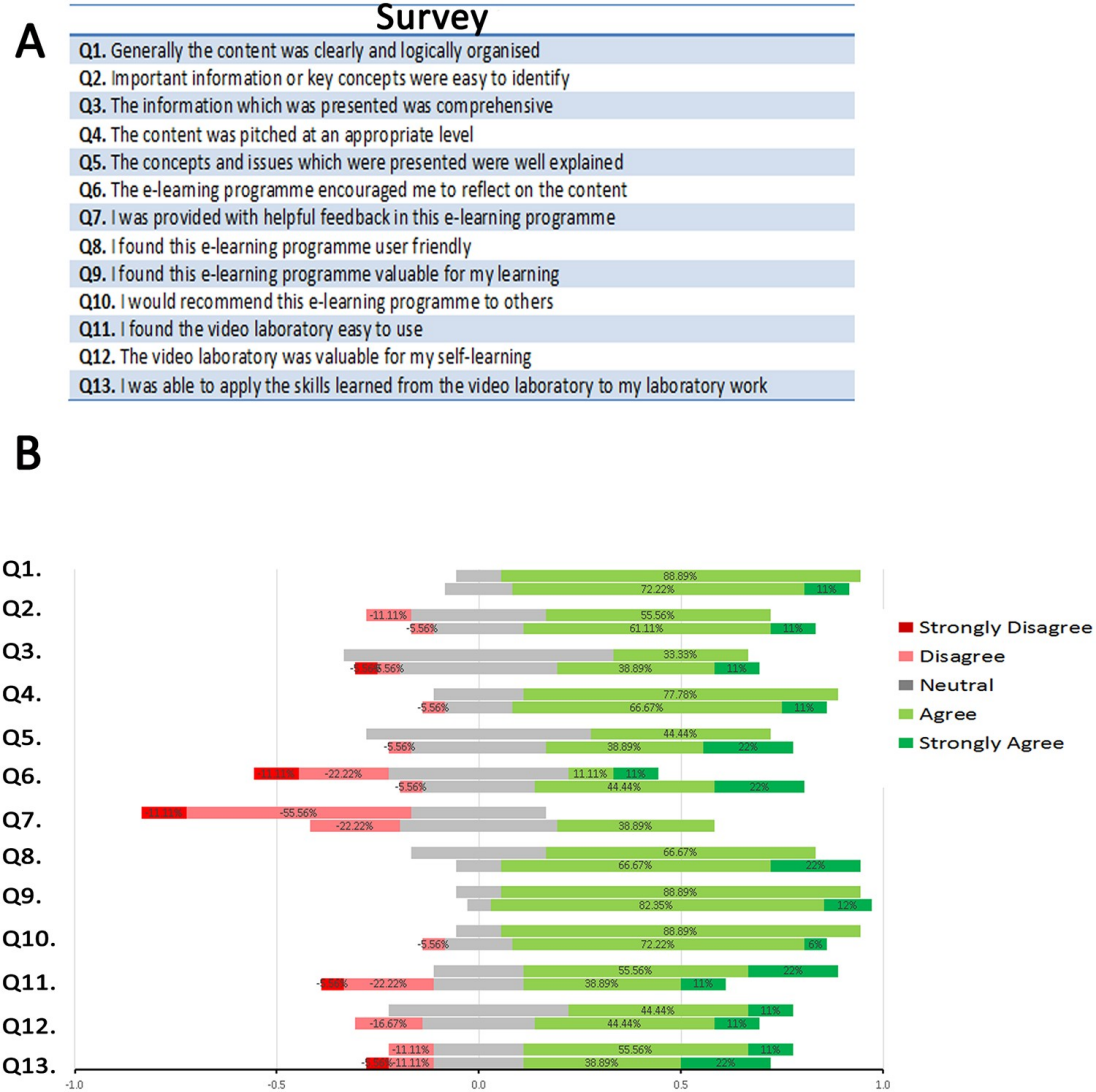
Response to the survey questions. The survey questions (A) and answers from users of the static website (first row) and interactive website (second row) categorized using a Likert scale (from strongly agree to strongly disagree) and plotted as a diverging stacked bar chart.

The survey was delivered at the end of the curricular year to the group of students that used the static website and to the group that used the interactive website. The survey was the same for both groups (Fig 2A). From classes of 57 (static website) and 55 students (interactive website), 38.6% and 58.2% participated in the paper based survey, respectively. All participants responded to all the questions in the survey (Fig 2B) choosing from 5 options (strongly disagree to strongly agree). There were 13 questions, and the median response for the groups was equal (4 (IQR = 1–5) for the static website and 4 (IQR = 1–5) for the interactive website. Responders commented positively (agree/strongly agree) that the content was clearly and logically organized (19/22 students (86.4%) for the static website and 26/32 students (81.2%) for the interactive resource). Only a small proportion of students in each group felt that important information/key concepts were not easy to identify (disagree: 3/22 (13.6% (for the static website and 2/32 or 6.3% for the interactive website). Users of the static website were mostly neutral, [median 3(IQR = 3–4)] about the resource being comprehensive, with none of them disagreeing with the comment. For the interactive website, users agreed and strongly agreed [median 4 (IQR = 1–5)] that the information was comprehensive but a small percentage disagreed and strongly disagreed (4/32 responses). Asked to comment if the information in the websites was delivered at the right level, the median response of the static website users was ‘agreed’ [median 4 (IQR = 3–4)] and so was the median response from users of the interactive website [4 (IQR = 2–5)] with only 2 students (6.25% of respondents) disagreeing with the statement. We asked students whether the information in the websites was well explained and the median response was ‘neutral’ 3 (IQR = 3–4) with 10/22 students or 45% of the static website users agreeing 4 (IQR = 2–5) and 19/32 (59%) of respondents about the interactive website agreeing/strongly agreeing.

Of the group of students with access to the static website, 22% disagreed (5/22) and 14% (3/22) strongly disagreed that the website encouraged them to reflect on the content of study (Survey Question 6). In comparison, responses from the group with access to the interactive website, showed no students strongly disagreed and only 6% (2/32) disagreed with this question. Many students with access to the static website felt that they did not get sufficient feedback via the website (strongly disagree/disagree total 67%; Question 7). Both groups found the e-learning experience to be user-friendly (agree/strongly agree total 19/22 (86.4%) for the static site users and 23/32 (71.9%) for the interactive site (Question 8) and valuable for their learning (Question 9). All respondents would be willing to recommend it to others, except for 5% of the interactive site responders (Question 10). In general, students evaluating the static website did not dislike the resource, but the interactive website has resulted in more positive comments about resources for self-learning and self-assessment.

Three questions in the survey asked about a specific laboratory video activity that was present in both the static and interactive websites. A total of 17/22 (77%) of respondents who used the static website agreed or strongly agreed and none disagreed that the laboratory video was easy to use. This compared with 16/32 (50%) of respondent who used the interactive site agreeing/strongly agreeing that the video was easy to use (Question 11). Asked about the value of the video for their self-learning, only users of the interactive website did not find it valuable (5/32 students; Question 12). In addition, while most of the responders agreed with the idea that they were able to apply the skills learnt from the video to their laboratory work, 3 responders with access to the static website and 5 responders with access to the interactive website disagreed/ strongly disagreed (Question 13).

### Focus group

The focus group questions were directed towards the user’s appreciation of the best and worst aspects of the website they used, the accessibility of the material it contained and the flexibility of its use. Recommendations were extracted from the focus groups and are listed in Table 2.

The websites were highly appreciated by the seven students in the focus group. For example, one student commented about the interactive website that *“…the laboratory website was really helpful because it helped you to re-look and re-think what you’ve done before*”. Four students that used the interactive website explained that they had expected to get a stronger indication from the educator of what content was examinable. The focus group participants indicated that the digital images and the main video provided for the laboratory sessions were the most memorable and meant that the demonstrator did not need to personally assist each student.

‘The videos were the best especially like the dissection of the cow’s eye …. and just having the video of that was very helpful and if we can have more videos that would be great.’

‘The images were really good … the videos and stuff like that for the pig dissection lab especially were really helpful because it was really hard for all of the demonstrators to get round to everyone and so that was really informative’.

The interactive website was praised because “…*at each laboratory you had these questions on the internet as well*, *on the e-learning site*, *which kind of indicated your learning objectives and exactly what you need to know and what you didn’t need to know so you didn’t have to like waste time in the process of doing it”*. Students in both groups also recognized that the video and the digital images were especially important for visual learners: “…*because this provided a good overview of the structure of the eye*. *It is good to actually see the parts of the eye that we have only drawn or visualized cartoons of*. *Seeing the actual layers/structures themselves along with the refresher description on the right [of the webpage] is very beneficial*.” The repeating ideas that emerged from the groups were: *using the website promoted deeper understanding of concepts* and *time with the instructor to discuss and follow-up questions is needed*. A comment on accessing the material by a user of the static website was: *“It’s just that we had the old website and we had to go through a few clicks to get to it”*. Overall a higher proportion of students using the interactive website felt that the site was better organized and directed towards the activity of the day: “*It was more helpful because the information we were questioned on was provided in iViewer*, *rather than just being provided with descriptions of the layers (tissue layers) in the static website*”.

When prompted to comment on what they did do when there were doubts about the content and when would they clarify these doubts, students had comments matching this response: *“I did*, *right before the test”*.

## Discussion

This study has identified that e-learning resources significantly contribute to blended teaching of the anatomy and physiology courses in Optometry. Students were positive about the value of the website for their learning and the clarity of the website content, and the possibility of receiving feedback via the interactive website. However, this e-learning activity did not influence the pass rates. In the focus group, students reported they preferred to have the teacher in class over the website alone as they felt the need to revise content with the educator before the test. There is acceptance of online learning methods but there is still dependence on the educator as the main administrator of their learning.

The creation of an e-learning resource (either static or interactive) for basic education in eye anatomy has allowed us to standardize the basic biological knowledge we impart to the students. Our results show that the introduction of an interactive version of the website did not significantly improve students’ laboratory test pass rate but did significantly contribute to a positive student experience and their transition to a practical model that interlinked academic support and self-directed learning [26].

It is interesting to note that, even though a greater proportion of students rated the interactive website positively, the addition of more learning materials and resources did not change laboratory average marks. Only the users of the interactive website had a small numerical gain in test results following the dissection of the eye video. The interactive website may have demanded more time and required higher performance, perhaps considered by some students as detrimental to the learning environment at the expense of reducing contact with the educator. This may have affected the comparison and the perceived undervalue of the interactive website by some students. At the time of the survey, all students had completed the task evaluated here which was run under the same settings and by the same educator. Imperfect recall of the experience is to the best of our knowledge the only possible factor that may have been a limitation in the study.

We asked about the effectiveness of small group learning in an Optometry class [27] and the impact of modifying the teaching process on student learning. Active learning approaches have significant benefits in teaching Science, Technology, Engineering and Mathematics (STEM) subjects [28], where students in traditional lecture-focused classes are 1.5 times more likely to fail than students in active learning class environments [29]. Moreover, we sought to enhance equal education opportunities for Optometry students [12, 30, 31] coming from a diversity of educational backgrounds by allowing students to control content acquisition at a speed, time and place that was convenient to them. Whilst learners were encouraged to be independent and to actively seek online information and support [32] the inclusion of online quizzes allowed students to self-assess understanding at the end of each module. Optometry students were generally positive about their e-learning experiences. The focus group responses significantly contributed to our evaluation of a blended environment, as we did not predict that teacher-student interaction would remain an important component in an online environment [33]. We believe that this is somehow related to the students’ expectation of some form of instructive feedback delivered by an educator on any activity they conduct. Conversely, it appeared that self-assessment quizzes at the end of each laboratory activity were appreciated as feedback by the majority of users of the interactive website, as only 22% of students did not find these helpful. The online quiz activities provided an option at the end of exercises to check the correct answer, allowing students to identify any areas of weakness and remediate them before summative assessment. Despite this, we found that the interactive laboratory website was not associated with a significant improvement in laboratory test marks, unlike other studies which have found a significant improvement in test marks following introduction of online formative feedback quizzes [34, 35]. Since the study of the Anatomy and Physiology of the eye is being undertaken by students who already have a strong motivation to understand ocular anatomy, we conclude that the website alone is not a contributor to student’ marks. Nevertheless, in their end of year course evaluation students exposed to the interactive website listed the online resources as one of the most helpful tools that enhanced their learning.

The focus groups identified that within the blended teaching environment tutor or teacher engagement and individualized student support are still major factors that influences the success of online learning experiences [36]. Student feedback emphasized the importance of teacher presence to set parameters, facilitate discourse and focus discussions to ensure learning objectives and outcomes were achieved. To be effective, the facilitator must have specialist knowledge of the topic, a willingness to be involved in students’ learning and good communication skills [37, 38]. Only teaching assistants with good knowledge of the examinable material, as well as training in facilitating laboratory-based teaching were invited to participate within our course. Facilitators were encouraged to take a student-centered approach and facilitate collaborative knowledge construction, rather than simply providing information [39, 40]. The inclusion of formative online quizzes helped learners take control of their own learning [41] as well as providing the e-facilitator with a method to monitor student progress [42]. Where obvious deficits in knowledge occurred, students could be re-directed to the appropriate learning material(s) or additional resources provided to help overcome weaknesses. Anatomy and physiology of the eye is not a complex topic but, depending on how key concepts are integrated into the curriculum, students might have different interpretations of syllabus requirements. Our results suggest that an online learning system alone does not provide for better guidance of learning objectives, and teacher-guided methods highlighting specific learning outcomes were preferred. We have addressed this requirement, by providing Teaching Assistants and Tutors who have experience in the topics, but who also have different backgrounds (not only Optometry) to allow the laboratory environment to be a forum of integrative learning. This Anatomy and Physiology course also employs Senior Optometry and Graduate Optometry students who can guide the activities and share their experiences while addressing learning objectives.

The focus group students indicated that they primarily used the websites before the examinations, to review their knowledge, rather than to prepare for the laboratory sessions. Although this does not seem to be aligned with the purpose of introducing an online activity in the class, from the educator perspective, less time was needed for explaining activities during the laboratory session which allowed more time to focus on answering questions and expanding on basic knowledge rather than talking through the mechanics of the activity. The next step is to combine application of this knowledge with current evaluation techniques such as those that require students to draw and sketch diagrams to explain the anatomy of the tissue. This approach provides an element of interactivity that students appreciate because it enables them to test their own understanding.

This study used paper-based survey. Our first attempt at collecting data was aligned with the University proposal to migrate to an exclusively on-line evaluation system, and we implemented online questionnaires. However, students did not respond (only 1%) to the online survey, preferring to engage in a paper-based format. Other studies have identified a poorer response rate in online versus paper-based surveys [43], and our study showed a similar lack of interest in participating online. While there is increasing support for online surveys [43, 44] there are studies that find lower responses than for paper based surveys when students are not offered a small monetary or grade incentive [45].

In summary, we have investigated student performance and obtained feedback on preference for traditional, teacher-based methods using a static website and compared those with preference for a blended environment including interactive online learning and evidence-based practice in curricular activities. Although access to the interactive website did not significantly improve students’ grades, the responses of students who had access to the interactive website were positive, although critical comments were directed towards the perceived absence of the educator assistance when the online tool was used. We concluded that in the teaching of Anatomy and Physiology in the Optometry programme a blended style that includes the educator as the main administrator of student learning is necessary.
